# 166 Do Local Sports Partnerships’ physical activity programs for middle-aged adults and older adults in Ireland work?

**DOI:** 10.1093/eurpub/ckae114.059

**Published:** 2024-09-26

**Authors:** Enrique Garcia Bengoechea, Catherine Woods

**Affiliations:** Physical Activity for Health Research Centre, Health Research Institute, University of Limerick, Limerick, Ireland; Department of Physical Education and Sport Sciences, University of Limerick, Limerick, Ireland; Physical Activity for Health Research Centre, Health Research Institute, University of Limerick, Limerick, Ireland; Department of Physical Education and Sport Sciences, University of Limerick, Limerick, Ireland

## Abstract

**Purpose:**

To strengthen the practice-based evidence needed to complement the more traditional evidence-based practice, pragmatic, yet robust, evaluations of real-world programs are necessary. This study sought to add to the existing evidence for the effectiveness of physical activity (PA) programs for middle-aged and older adults offered by public Local Sports Partnerships (LSP) in Ireland.

**Methods:**

Using a cluster randomised control trial, we collected and analysed data from 463 individuals aged 50+ years, who participated in the Move for Life (MFL) study. Outcomes were self-reported compliance with PA guidelines (PAGL); accelerometer-based moderate-to-vigorous intensity PA (MVPA), light intensity PA (LiPA), standing time and sedentary time; body composition, physical function and psychological wellbeing. LSP programs included Bike for Life, Women on Wheels, Go for Life Games, Get Ireland Walking and Men on the Move. Mixed effects regression models to assess Group x Time interactions over a 6-month period, while accounting for repeated measurements, relevant confounders in a social ecological framework and the effects of the MFL intervention against a control group.

**Results:**

When combining the two cycling programs, positive effects on meeting PAGL for all programs (all p<.05) was found. Considering accelerometer-based PA, evidence emerged for positive program effects on MVPA (Bike for Life + Women on Wheels, Get Ireland Walking), LiPA (Bike for Life, Go for Life), and sedentary time (Bike for Life + Women on Wheels, Go for Life) (all p<.05). No evidence of program effects on body composition (BMI, waist circumference) was found. Regarding physical function, while there were no significant differences between groups for Timed Up and Go; participants in Bike for Life + Women on Wheels and Get Ireland Walking outperformed their control group counterparts in the Six-Minute Walk Test (p<.01). Lastly, Men on the Move was the only program where we observed a positive trend over time in wellbeing scores compared to control participants (p=.087).

**Conclusions:**

Despite sample size limitations, the results show the effectiveness of LSP programs, notably in terms of energy expenditure outcomes, while highlighting areas for improvement regarding outcomes related to body composition, physical function and, particularly, psychological wellbeing.

**Funding Source:**

Grant HaPAI/2017/CW

